# Protocol to identify protein-protein interaction networks in *Solanum tuberosum* using transient TurboID-based proximity labeling

**DOI:** 10.1016/j.xpro.2023.102577

**Published:** 2023-09-20

**Authors:** Li Shi, Tatiana Marti Ferrando, Sergio Landeo Villanueva, Matthieu H.A.J. Joosten, Vivianne G.A.A. Vleeshouwers, Christian W.B. Bachem

**Affiliations:** 1 Laboratory of Plant Breeding, Wageningen University & Research, Droevendaalsesteeg 1, 6708 PB Wageningen, the Netherlands; 2 Laboratory of Phytopathology, Wageningen University & Research, Droevendaalsesteeg 1, 6708 PB Wageningen, the Netherlands

**Keywords:** Plant Sciences, Molecular Biology, Protein Biochemistry

## Abstract

Protein-protein interactions (PPIs) in crop plants remain largely unexplored. Here, we provide a protocol for identifying PPIs in potato (*Solanum tuberosum*) using TurboID-mediated proximity labeling. We transiently expressed constructs for a nucleus-located transcription factor and a plasma membrane-localized receptor-like kinase fused to TurboID to identify PPIs in potato leaves. We describe the plasmid construction, plant material, agroinfiltration, biotin treatment, protein isolation, free biotin removal, western blot analysis, and enrichment of biotinylated proteins for mass spectrometry analysis.

## Highlights

• TurboID-enabled biotin-based proximity labeling protocol for *Solanum tuberosum*
• Application of TurboID-PL in nuclear and membrane-bound proteins
• A detailed protocol from preparing plants material and transgene design to protein identification
• Visualization of biotinylated proteins by western blotting

## Abstract (editor highlights)

Publisher’s note: Undertaking any experimental protocol requires adherence to local institutional guidelines for laboratory safety and ethics.

## Graphical abstract

**Figure:**
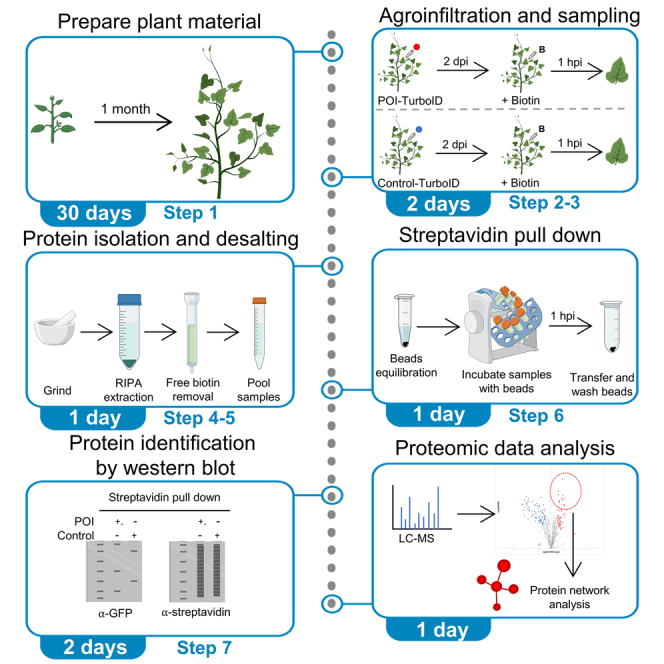

## Before you begin

### Experimental design considerations

Selection of genotypes*:* This protocol describes the procedure for transient TurboID-based proximity labeling (PL) of the potato transcription factor CYCLING DOF FACTOR1 (StCDF1) in CE3027 and the receptor-like kinase SUPPRESSOR OF BIR1 of *Nicotiana benthamiana* (NbSOBIR1) in MCD-321. CE3027 is an offspring plant of the diploid C x E population and it was selected to study the role of StCDF1 in tuberization in previous research.^1^^,^^2^ MCD-321 is an offspring plant of a cross between the wild potato plants *Solanum microdontum* subsp*. microdontum* and an F1 clone from a *S. microdontum* subsp*. gigantophyllum* x *S. verrucosum* cross.^3^

Selection of experimental controls: Design the control TurboID-construct based on the localization of your protein bait of interest. The proteins used in this study are localized either in the nucleus (StCDF1) or in the plasma membrane (NbSOBIR1). As a control, the fusion protein YFP-YFP-TurboID^4^^,^^5^ was used. The YFP-YFP-TurboID is located at both nucleus and cytoplasm, which has been previously reported by Kim et al*.*^4^

Experimental test: Before starting the procedure of the potato PL-mass spectrometry (MS) protocol, we recommend testing the TurboID fusion constructs by transient expression in *N. benthamiana leaves*, as it is an easy plant model to work with. Then, we suggest testing them in the potato genotype of interest. Additionally, we suggest to co-express the labeled bait with a known interactor to confirm that this interactor is being biotinylated. The selected interactor protein should also be tagged with a different label than the bait protein, to allow its specific detection on western blots. In potato, it is advisable to infiltrate biotin and to collect leaf samples at different time points (e.g., at 1, 3 and 6 h post infiltration (hpi) of biotin), as well as including a sample without biotin. Both *N. benthamiana* and potato tests can be performed with 2 g of leaf tissue following the same steps as for MS sample preparation. Per test sample, only one PD-10 desalting (steps 23–28) column and 50 μL of streptavidin bead suspension (steps 29–36) is needed. After the last wash of beads with radioimmunoprecipitation assay (RIPA) buffer (step 36), proteins are eluted with 80 μL 4× Laemmli Sample Buffer (Bio-Rad) diluted in RIPA and checked on Western blot with the appropriate antibodies (steps 39–52).

Identification of biotinylated proteins: After the pull down with streptavidin beads, the biotinylated proteins are pre-processed depending on the approach that is going to be used. In most cases, peptides are obtained from an on-bead digestion using trypsin. Peptides are subsequently separated by liquid chromatography (LC) prior to identification by MS. We do not describe these steps in detail as they differ for each MS facility.

### Plasmid construction

**Timing: minimum 10 days**

The process involves amplifying the DNA sequence of the gene that encodes the protein of interest (POI), followed by gel extraction or PCR product purification, performing Gateway cloning, transforming *Escherichia coli*, and purifying the plasmid.

1. Design primers to build the plasmids for Gateway cloning.

***Note:*** The reverse oligonucleotide used for PCR in the DNA sequence of the genes had the stop codons removed. The PCR fragment is cloned in a pEG101-TurboID (TurboID) vector for C-terminal tagging with YFP::V5::TurboID::HA5. If the gene under investigation is known to have its protein binding domain at the C-terminal end, consider tagging the gene at the N-terminal end. Generating such a vector based on the ImpGWB series should be unproblematic.

***Note:*** In this protocol we used primers with attB ends to perform a BP reaction to the Entry vector pDONR221 (attP). After BP reaction, the pDONR221 construct contains the gene flanked with attL sites that will allow the LR reaction to pEG101-TurboID (attR).

2. Clone DNA sequence of the gene in the TurboID vector.
  a. Prepare the PCR reaction mix to generate a Gateway-compatible PCR product with attB ends.
  b. Visualize the PCR products in 1% agarose gel electrophoresis.
  c. Purify PCR products with QIAquick Gel Extraction Kit or Zymo DNA Clean & Concentrator Kit.
    ***Note:*** If a single band is visualized at the expected size, perform a PCR product purification using Zymo DNA Clean & Concentrator Kit. When there are bands in addition to the target-sized DNA fragments, perform gel purification of the desired fragment using the QIAquick Gel Extraction Kit, according to the manufacturer’s instructions (https://www.qiagen.com/us/resources/resourcedetail?id=a72e2c07-7816-436f-b920-98a0ede5159a&lang=en).
  d. Perform a BP recombination reaction with Invitrogen Gateway BP Clonase II Enzyme mix between the PCR product and the pDONR221 vector (Invitrogen Gateway pDONR221 Vector) to create the Entry vector.
  e. Transform BP reaction mix in One Shot TOP10 Chemically Competent *E. coli* cells, according to manufacturer’s instructions (https://www.thermofisher.com/document-connect/document-connect.html?url=https://assets.thermofisher.com/TFS-Assets%2FLSG%2Fmanuals%2Foneshottop10_man.pdf).
  f. Pick three single colonies into LB medium containing 50 μg/mL kanamycin.
  g. Incubate cultures for 18 h at 37°C in the shaker at 200 rpm.
  h. Purify plasmids from the transformed cells using the QIAprep Spin Miniprep Kit following the manufacturer’s instructions. (https://www.qiagen.com/us/resources/resourcedetail?id=22df6325-9579-4aa0-819c-788f73d81a09&lang=en).
  i. Verify the sequence of Entry vectors using the primers M13-Fw and M13-Rv for Sanger sequencing.
  j. Perform a LR recombination reaction with Invitrogen Gateway LR Clonase II Enzyme mix between the verified Entry vector and the Destination vector to create an Expression vector.
    ***Note:*** The Destination vector used in this study was generated by Kim et al.^4^^,^^5^ with CaMV 35S promoter (p35S). To improve the biotinylation efficiency, it may be beneficial to consider changing the promoter in the Destination vector based on the POI's expression pattern, timing, and tissue. For example, replacing the p35S with a SUC2 promoter to study phloem specific PPIs. If the protein is naturally well expressed and accumulated in potato plants, consider the use of the native promoter.
  k. Transform LR reaction mix in One Shot TOP10 Chemically Competent *E. coli* cells, as described above in *steps e-h.*
  l. Verify the sequence of the Expression vector using the primers P35S-CaMV-Fw and YFP-Rv.
  m. Store the Expression vector in Milli-Q water or the QIAprep Spin Miniprep Kit elution buffer at −20°C until further use.

## Key resources table

**Table:** 

REAGENT or RESOURCE	SOURCE	IDENTIFIER
**Antibodies**		

Goat anti-rabbit IgG, HRP (1:10000)	Agrisera	AS09-602
BirA (mutated/TurboID) (1:5000)	Agrisera	AS20-4440
Streptavidin protein, HRP (1:5000)	Thermo Fisher Scientific	21124
Anti-GFP, HRP (1:1000)	Invitrogen	A10260

**Chemicals, peptides, and recombinant proteins**		

Chloramphenicol (Cm)	Duchefa Biochemie	4800-94-6
Carbenicillin (Cb)	Duchefa Biochemie	56-75-7
Kanamycin (Kn)	Duchefa Biochemie	25389-94-0
Tetracycline (Tet)	Duchefa Biochemie	64-75-5
Rifampicin (Ra)	Duchefa Biochemie	13292-46-1
Acetosyringone (3′,5′-dimethoxy-4′-hydroxyacetophenone)	Sigma-Aldrich	D134406
MES monohydrate (2-(N-morpholino)-ethane sulfonic acid)	Duchefa Biochemie	145224-94-8
Murashige and Skoog medium including vitamins	Duchefa Biochemie	M0222
Murashige and Skoog medium basal mixture	Duchefa Biochemie	M0221
10× Tris/glycine/SDS	Bio-Rad	1610772
StartingBlock (TBS) Blocking Buffer	Thermo Scientific	37542
Precision Plus Protein WesternC Blotting Standards	Bio-Rad	1610376
4× Laemmli sample buffer	Bio-Rad	1610747
Biotin	Merck	B4639-100MG
Pierce Protease Inhibitor Tablets, EDTA-free	Thermo Scientific	A32965
MG-132, proteasome inhibitor	Sigma-Aldrich	M7449-1ML

**Critical commercial assays**		

QIAquick Gel Extraction Kit (50)	QIAGEN	28704
DNA Clean & Concentrator-5	Zymo Research	D4004
Invitrogen Gateway BP Clonase II enzyme mix	Thermo Scientific	11789020
Invitrogen Gateway LR Clonase II enzyme mix	Thermo Scientific	11791020
QIAprep Spin Miniprep Kit (50)	QIAGEN	27104
Thermo Scientific Pierce Streptavidin Magnetic Beads	Thermo Scientific	10615204
Disposable PD 10 desalting columns	Sigma-Aldrich	GE17-0851-01
Trans-Blot Turbo Mini 0.2 μm PVDF Transfer Packs	Bio-Rad	1704156
SuperSignal West Dura Substrate	Thermo Scientific	34076

**Experimental models: Organisms/strains**		

One Shot TOP10 Chemically Competent *E. coli* cells	Thermo Scientific	C404010
AGL1 (Cb^R^, Cm^R^)	Petti et al.^6^	N/A
C58C1 (Te^R^, Ra^R^)	N/A	N/A

**Recombinant DNA**		

pEG101-YFP::V5::TurboID::HA (Kn^R^)	Kim et al.^4^^,^^5^	N/A
YFP-YFP::V5::TurboID::HA (Kn^R^)	Kim et al.^4^^,^^5^	N/A
NbSOBIR1-YFP::V5::TurboID::HA (Kn^R^)	This paper	N/A
StCDF1-YFP::V5::TurboID::HA (Kn^R^)	This paper	N/A
Invitrogen Gateway pDONR221 Vector	Thermo Scientific	12536017

**Oligonucleotides**		

M13-Fw: GTAAAACGACGGCCAG	This paper	N/A
M13-Rv: CAGGAAACAGCTATGAC	This paper	N/A
P35S-CaMV-Fw: CTATCCTTCGCAAGACCCTTC	This paper	N/A
YFP-Rv: AAGAAGATGGTGCGCTCCTG	This paper	N/A

**Other**		

Screw cap tube, 15 mL, (LxØ): 120 × 17 mm, PP, with print (V-shaped 15 mL tubes)	Sarstedt	62.554.502
Eppendorf Protein LoBind tubes 2 mL (2 mL LoBind tubes)	Eppendorf	EP0030108132-100EA
Eppendorf Safe-Lock tubes 1.5 mL	Eppendorf	0030120086
Gene Pulser/MicroPulser electroporation cuvettes, 0.1 cm gap	Bio-Rad	1652089
MicroPulser electroporator	Bio-Rad	1652100

## Materials and equipment

• Chloramphenicol (Cm) 50 mg/mL: dissolve 500 mg of Cm in 10 mL of ethyl alcohol pure. Store at -20°C in 1 mL aliquots. Dilute stock to 25 μL/mL in specified culture media.
• Carbenicillin (Cb) 50 mg/mL: dissolve 500 mg of Cb in 10 mL of Milli-Q water and filter sterilize through 0,2 μm filter. Store at −20°C in 1 mL aliquots. Dilute stock to 50 μL/mL in specified culture media.
• Kanamycin (Kn) 50 mg/mL: dissolve 500 mg of Cm in 10 mL of Milli-Q water and filter sterilize through 0,2 μm filter. Store at −20°C in 1 mL aliquots. Dilute stock to 50 μL/mL in specified culture media.
• Tetracycline (Te) 15 mg/mL: dissolve 150 mg of Cm in 10 mL of methanol. Store at −20°C in 1 mL aliquots. Dilute stock to 15 μL/mL in specified culture media.
• Rifampicin (Ra) 50 mg/mL: dissolve 500 mg of Cm in 10 mL of DMSO. Store at −20°C in 1 mL aliquots. Dilute stock to 50 μL/mL in specified culture media.
• Streptavidin Protein, HRP 1 mg/mL: add 2 mL of Milli-Q water to the tube that contains 2 mg of protein. Store at −20°C in 50 μL aliquots.
• **Acetosyringone** (4-hydroxy3,5-dimethoxyacetophenone) 200 mM: dissolve 196 mg of acetosyringone in 5 mL of DMSO. Store at −20°C in 0.5 mL aliquots. Dilute stock to 200 μM in the specified culture media.

***Note:*** Store at −20°C in 0,5 mL aliquots.

• **MES solution 1:** (2-(N-morpholino)-ethane sulfonic acid) 1 M, pH 5,6: dissolve 4,265 g of MES in 20 mL of Milli-Q water, adjust with NaOH to pH 5,6.

***Note:*** MES is difficult to dissolve. First add 10 mL of Milli-Q, adjust with NaOH to pH 5,6 and then adjust the final volume to 20 mL. Filter sterilizes through 0.2 μm filter. Store at −20°C in 2 mL aliquots.

• **MES solution 2:** (2-(N-morpholino)-ethane sulfonic acid) 10 mM, pH 8: dissolve 32 mg of MES in 15 mL of Milli-Q water, adjust with NaOH to pH 8.

***Note:*** 10 mM MES for dissolving biotin should be prepared before use. Keep MES in the dark by covering the bottle or tube with aluminum foil.

• MG-132 10 mM: MG-132 (M7449-1ML) is a ready-made solution at 10 mM in DMSO. Dilute to 40 μM in specified buffer.
• **Biotin** 10 μM: dissolve 0.37 mg of biotin in 150 mL of MES solution 2 (10 mM).

***Note:*** Prepare fresh on the day of use. This volume (150 mL) is calculated for a set of 15 plants, using 3 leaves per plant and a surface area of each of the leaves of about 30 cm^2^. MCD-321 has leaves with a surface area of 30 cm^2^ approximately each and CE3027 has leaves of approximately 15 cm^2^ each.

***Optional:*** Add proteasome inhibitor MG-132 40 μM to biotin 10 μM solution to reduce the degradation of ubiquitin-conjugated proteins before collection of samples.

**Table:** MS20 plant propagation media

Reagent	Final concentration	Amount
Murashige and Skoog medium including vitamins	4.4 g/L	4.3 g
Sucrose	20 g/L	20 g
ddH_2_O	N/A	1 L
Adjust to pH 5.8 with 1 M NaOH	N/A	N/A
Micro agar	8 g/L	8 g

***Note:*** Autoclave at 121°C for 20 min. Pour media immediately after autoclaving. Keep MS20 agar for max. 6 months at 20°C–25°C. Grow plants in MS20 for max. 6 months.

**Table:** LB (Lysogeny broth) media

Reagent	Final concentration	Amount
Tryptone	10 g/L	10 g
Yeast extract	5 g/L	5 g
NaCl	10 g/L	10 g
ddH_2_O	N/A	1 L

***Note:*** Autoclave at 121°C for 20 min. Keep LB media for max. 1 year at 20°C–25°C.

**Table:** YEB (yeast extract beef) media

Reagent	Final concentration	Amount
Beef extract	5 g/L	5 g
Bacteriological peptone	5 g/L	5 g
Sucrose	5 g/L	5 g
Yeast extract	1 g/L	1 g
MgSO_4_·7H_2_O	0.492 g/L	0.492 g
ddH_2_O	N/A	1 L

***Note:*** Autoclave at 121°C for 20 min. Keep YEB media for max. 1 year at 20°C–25°C.

**Table:** MMA buffer

Reagent	Final concentration	Amount
Sucrose	20 g/L	10 g
Murashige and Skoog basal salts	5 g/L	2.5 g
MES	1.95 g/L	0.98 g
ddH_2_O or Milli-Q	N/A	0.5 L
Adjust to pH 5,6 with 1 M NaOH	N/A	N/A
Acetosyringone	200 μM	0.5 mL

***Note:*** Add reagents in the provided order. Add the acetosyringone after pH adjustment. Make fresh in ddH_2_O or Milli-Q on the day of use.

**Table:** RIPA (Radioimmunoprecipitation assay) buffer

Reagent	Final concentration	Amount
Tris-HCl pH 7.6	25 mM	3.03 g
NaCl	150 mM	8.76 g
Sodium deoxycholate	1% (w/v)	10 g
SDS	0.1% (w/v)	1 g
NP-40	1% (v/v)	10 g
ddH_2_O	N/A	1 L

***Note:*** RIPA can be stored at 4°C for a month without protease and proteasome inhibitors. Add 1 tablet of protease inhibitor cocktail and MG-132 (40 μM) per 50 mL of RIPA the day of use when specified in the protocol.

**Table:** NP-40 free RIPA buffer

Reagent	Final concentration	Amount
Tris-HCl, pH 7.6	25 mM	3.03 g
NaCl	150 mM	8.76 g
Sodium deoxycholate	1% (w/v)	10 g
SDS	0.1% (w/v)	1 g
ddH_2_O	N/A	1 L

***Note:*** NP-40 free RIPA can be stored at 4°C for a month without protease and proteasome inhibitors. Add 1 tablet of protease inhibitor cocktail per 50 mL of NP-40 free RIPA the day of use when specified in the protocol.

**Table:** TBS 10×

Reagent	Final concentration	Amount
Tris-HCl pH 7.5	200 mM	12 g
NaCl	150 mM	4 g
ddH_2_O	N/A	0,5 L

***Note:*** Store TBS 10× for max. 3 months at 4°C. Make TBS 1× with ddH_2_O on the day of use and add 0.1% Tween 20 to make TBS-T 1×. Store TBS-T 1× for max. 1 month at 4°C.

## Step-by-step method details

### Propagating plant material

**Timing: 30–35 days**

The desired potato genotypes are propagated in appropriated media under controlled conditions before growing them in the greenhouse for transient expression assays. After this step, potato plants are ready to be transiently transformed with *Agrobacterium tumefaciens* carrying the TurboID construct.

1. Grow fresh cuttings of potato plants on MS20 *in vitro* at 24°C in a climate chamber, under long day conditions (16 h/8 h day/night), for 2 weeks.
2. Transfer plantlets to 5 cm diameter round pots containing sterilized potting soil in climate-regulated greenhouse compartment.
3. Grow for 1 week within a temperature range of 18°C–22°C and long day conditions.
4. Transfer to 11 × 11 cm square pots or to 14 cm diameter round pots.
5. Grow for 3–5 weeks under the same conditions.

***Note:*** Some potato genotypes grow better in round pots. Check troubleshooting – problem 2.

***Note:*** Number of plants to grow depends on setting of the experiment. Samples for MS undergo triplicate sending for statistical analysis. One replicate consists of a set of five plants and per plant, 3–5 leaves are infiltrated with the construct. Therefore, 15 plants in total per set of TurboID construct (5 × 3).

### *Agrobacterium tumefaciens* preparation and agroinfiltration

**Timing: 1–2 weeks**

TurboID constructs are transformed into *Agrobacterium tumefaciens* and transiently transform on potato leaves with an appropriated transformation buffer.

6. Transform 100 ng of the plasmid containing the TurboID fusion constructs to 20 μL of electro-competent *Agrobacterium tumefaciens* (in 1,5 mL Eppendorf tube).
  a. Pipette *Agrobacterium* cells with DNA into a prechilled 0,2 cm electroporation cuvette.
  b. Pulse cells at 1,4 kV with MicroPulser Electroporator.
  c. Add 0,25 mL of YEB (or LB) media to the cuvette, mix, and immediately transfer cells into a clean 1,5 mL Eppendorf tube.
  d. Grow for 2 h at 28°C and 200 rpm.
  e. Plate 20–50 μL on LB agar plates (90 mm diameter) with the appropriate antibiotics.

***Note:*** In this protocol, YFP-TurboID and NbSOBIR1-TurboID constructs were transformed into *Agrobacterium* strain C58C1 and StCDF1-TurboID into AGL1. The antibiotics for C58C1 are Ra and Te, and for AGL1 are Cm and Cb. In addition, Kn antibiotic is added for selection of *Agrobacterium* colonies transformed with TurboID construct. Working concentrations are specified in “Materials and equipment”.

***Note:****A. tumefaciens* transformation by electroporation has high efficiency and usually produces 30–60 colonies using this set-up. Longer incubation times (up to 4 h) in LB will increase efficiency.

***Optional:*** Test different *A. tumefaciens* strains to check the accumulation of the fusion POI upon transient expression. Check troubleshooting – problem 1 & 5.

7. Pick a single transformed *A. tumefaciens* colony and grow in 10 mL of LB media with the appropriate antibiotics
8. Incubate for 2 days at 28°C with continuous shaking at 200 rpm.

***Note:*** We recommend verifying constructs transformed in *Agrobacterium*. Perform colony PCR with 5 μL of the *A. tumefaciens* culture using the primers P35S-CaMV-Fw and YFP-Rv, or a combination of the P35S-CaMV-Fw primer with a reverse primer from the gene. Send PCR product for Sanger sequencing with primers used for colony PCR.

9. Transfer the cultures to a 250 mL flask containing YEB media with the appropriate antibiotics and supplemented with acetosyringone (final concentration 200 μM) and MES solution 1 (final concentration 10 mM).
10. Incubate for 1 day at 28°C with continuous shaking at 200 rpm.
11. Collect the cells by centrifugation at 3,000 × *g* for 10 min.
12. Resuspend the pelleted cells in freshly prepared MMA buffer to an OD_600_ of 0,3–0,4.

***Note:*** For co-infiltration of two constructs, mix the *A. tumefaciens* cultures in a 1:1 ratio.

***Note:*** 30–50 mL of *A. tumefaciens* culture is usually enough for infiltrating 3–5 leaves per set of triplicates. Potato leaf size varies a lot and different genotypes have different structure, such as trichome density etc., which results in different requirements for *A. tumefaciens* infiltrate. On average, 1 mL is sufficient for 1 potato leaf.

13. Incubate the *A. tumefaciens* suspensions in MMA for 1–2 h at 20°C–25°C in dark without rolling or shaking before infiltration.
14. Fully infiltrate 3–5 leaves of each potato plant with the suspension from the lower side of the leaves with a 1 mL needleless syringe.

***Optional:*** Water the potato plants 1–2 h before agroinfiltration to facilitate the infiltration of the suspension, as the stomata will open. Make a scratch on the lower epidermis of the leaves to be infiltrated with a needle or scalpel to facilitate the infiltration. Check troubleshooting – problem 3.

***Note:*** Choose young, healthy, and just fully expanded leaves for the agroinfiltrations.

***Note:*** We recommend changing gloves between each set of constructs to avoid possible contamination between different constructs.

***Note:*** Infiltrated plants are maintained in the greenhouse within a temperature range of 18°C–22°C and under long day conditions.

### Leaf sample collection

**Timing: 2 days**

The recombinant TurboID bait proteins should be successfully expressed in the leaves after 2 days. Leaves are harvested and prepared for protein extraction procedure.

***Optional:*** Test with and without the addition of biotin. Check troubleshooting – problem 4.

***Optional:*** After 36–48 hpi, infiltrate freshly prepared solution of 10 μM biotin in MES solution 2 and collect leaf samples at 1–3 hpi after the infiltration of biotin.

***Optional:*** To reduce the degradation of ubiquitin-conjugated proteins, 40 μM MG-132 can be additionally supply together with biotin solution.

***Note:*** The concentration and incubation time of biotin treatment was determined based on previous studies in *Arabidopsis* and *N. benthamiana*.^4^^,^^5^^,^^7^

15. After 36–48 hpi, cut the leaves off, remove the petiole and middle vein from the base with scissors.
16. Place in a 50 mL tube with V-shaped bottom and immediately place into liquid nitrogen.

***Note:*** We recommend cleaning the scissors used for removing the petiole and middle vein with ethanol 70% between different treatments.

**Pause point:** Keep leaves at −80°C, either the intact leaves or ground, in tubes.

17. Grind samples in liquid nitrogen using a mortar and pestle to a fine powder and weigh.

***Note:*** Make sure that the triplicates are all approximately of the same weight; 3–5 g ± 0.5 g.

### Protein extraction

**Timing: 2–3 h**

Total protein is extracted from the transiently transformed potato leaves.

18. Add 2 mL of RIPA buffer, supplemented with protease inhibitor cocktail and MG-132, per gram of ground leaf.

***Note:*** MG-132 is a proteome inhibitor and blocks the activity of proteasomes. It targets protein degradation differently from the protease inhibitor. For some proteins, such as DELLA,^8^ MG-132 is crucial for its stability. Therefore, we recommended to supply MG-132 in with RIPA buffer.

19. Keep samples on ice, while vortex-mixing for 15 min.
  a. Vortex-mixing each sample, while keeping samples cold by placing the samples on ice during the rest time.
20. Pellet the debris by centrifugation for 30 min at 4°C at maximum speed (RCF: 17,000 × *g*).

***Note:*** Make sure that the supernatant is clear, without remaining particles.

21. Transfer the supernatant to a pre-cooled V-shaped 15 mL tube.
22. Adjust all samples to 10 mL with RIPA buffer, supplemented with protease inhibitor cocktail and MG132.

***Note:*** Preferably use LoBind tubes for all steps.

***Note:*** The total volume of the samples should be around 10 mL.

***Note:*** Each *Cytiva Life Sciences* PD-10 desalting column can only hold 2.5 mL per column. Per sample, approx. 10 mL of isolated protein solution is obtained, so 4 columns are needed.

### Removal of free biotin from the protein extracts with *Cytiva Life Sciences* PD-10 desalting columns

**Timing: 1–2 h**

Biotin present in the total extract of proteins is filtered out to avoid the competition for binding the streptavidin beads used in the next step for enrichment of biotinylated proteins.

***Note:*** The following steps should be performed in 4°C room.

23. Equilibrate the PD-10 desalting columns.
  a. Place the column in the provided rack and place a container below to collect the flow-through.
  b. Remove the top cap.
  c. Cut the sealed end and allow the storage solution to drain away.
  d. Wash the column 3× with 4 mL of RIPA without protease inhibitor cocktail and without MG132.
  e. Wash the column 1× with 4 mL of RIPA with protease inhibitor cocktail and with MG132.
24. Add 2.5 mL of the protein extract to the equilibrated PD-10 desalting column.
25. Discard the flow-through.
26. Transfer the columns to another rack, containing V-shaped 50 mL tubes.
27. Elute the columns with 3,5 mL of RIPA, supplemented with protease inhibitor cocktail and MG132.
28. Combine the eluates from each triplicate in one tube.

### Pull down of the biotinylated proteins with *Thermo Scientific Pierce* streptavidin magnetic beads

**Timing: 3 h**

Pull-down methodology using streptavidin beads is employed to enrich biotinylated proteins. Biotin labelled proteins from each sample pool will be subsequent analyzed by Western blot and/or LC-MS for their identification.

***Note:*** Perform the following steps at 4°C.

***Note:*** In parallel, perform a test for checking the protein extract on a western blot.

29. Equilibrate the streptavidin beads.
  a. Resuspend the beads by pipetting and stirring before use.
  b. Pipette 200 μL of streptavidin beads (50% slurry) per sample in a 2 mL LoBind tube.
    ***Note:*** For the test sample, use 50 μL of streptavidin beads per sample (50% slurry).
    ***Note:*** The volume of the beads per sample (200 μL for MS sample and 100 μL for test sample in this protocol) can be adjust based on the volume of desalted protein extracts and incubation time.^5^^,^^9^
    ***Note:*** To estimate the total volume of beads necessary to process all the samples, including an extra volume of 5% to account for pipetting errors, follow this calculation:
    Totalvolumeofbeads=105%×(Numberofsamples×Volumeofbeadspersample)
    For example, if there are 5 samples, and each sample requires 200 μL of beads:
    Totalvolumeofbeads=1,05×(5×200μL)=1050μL.
  c. Place the tube into a magnetic stand for 3 min to collect the beads.
  d. Discard the supernatant.
  e. Wash the beads 3× with 1 mL of RIPA, supplemented with protease inhibitor cocktail and MG-132.
    ***Note:*** For each washing step, add the buffer and mix by inversion until the beads are completely resuspended, and let the tube stand for 3 min in the magnetic rack, after which the supernatant is discarded.
30. After the last wash add 100 μL of RIPA per sample, supplemented with protease inhibitor cocktail, to the equilibrated beads and transfer the 100 μL resuspended beads to each of the V-shaped 15 mL tubes containing the desalted protein extracts.

***Note:*** For the test sample, add 50 μL of RIPA per sample, supplemented with protease inhibitor cocktail.

31. Incubate the samples in a rotator for 1 h at 4°C, at 10 rpm.

***Note:*** You can extend the incubation up to 18 h (overnight) at 4°C, ensuring a gentle and continuous shake at 10 rpm.

32. Wash the beads.
  a. Place the tubes into a magnetic stand for 3 min to collect the beads.
  b. Discard the supernatants.

***Note:*** The supernatants can be collected (20–50 μL) and stored at −20°C for testing the binding efficiency at a later time point.

33. Add 1 mL of NP40-free RIPA, supplemented with protease inhibitor cocktail, and mix by inversion of the tubes.
34. Transfer the mixture to a 2 mL LoBind tube.
35. Place the tubes into a magnetic stand for 3 min to collect the beads.
36. Wash the beads 3× with 1 mL of NP40-free RIPA, supplemented with protease inhibitor cocktail. Follow the same procedure as indicated in step **32** to wash the beads.
37. The samples are now ready for MS pre-processing. Go to **step 53.**
38. The sample test can be processed for western blot following **step 39.**

**Pause point: Store** the enriched biotinylated proteins at −20°C before further processing for MS.

### Identification of biotinylated proteins by western blotting

**Timing: 4–5 h**

After the enrichment, verify biotinylated proteins through Western blot analysis. Visualize proteins using various antibodies to confirm bait expression and its capacity to biotinylate other proteins.

***Note:*** This step is only performed for the protein samples that will NOT be processed for MS

39. Resuspend the streptavidin beads to which the biotinylated proteins are bound in 60 μL of RIPA.
40. Add 20 μL of 4× Laemmli Sample Buffer (Bio-Rad), previously mixed 9:1 with β-mercaptoethanol as the manufacturer’s recommendation (https://www.bio-rad.com/webroot/web/pdf/lsr/literature/4006028.pdf).
41. Incubate at 95°C for 10 min, in a shaker at 300 rpm.
42. Place the tubes on ice for 2 min.
43. Spin the beads down at top speed in a microcentrifuge.
44. Load 15–25 μL of the supernatant on an SDS-PAGE gel.

***Note:*** To avoid pipetting the beads, leave the tubes in the magnetic rack,

45. Load 5 μL of the ladder Precision Plus Protein WesternC Blotting Standards.
46. Run the gel in an electrophoresis cell with 1× Tris/Glycine/SDS buffer.
47. Transfer the proteins to a Trans-Blot Turbo Mini 0.2 μm PVDF membrane in the Trans-Blot Turbo system at 25 V at 1,3 A for 7 min.
48. Block the membrane with 10 mL of StartingBlock (TBS) Blocking Buffer, supplemented with 0.5% (v/v) of Tween 20 for 30 min.

***Note:*** The standard blocking buffer, which consists of 5% of fat-free milk powder in TBS-T 1×, contains biotin that potentially interferes with the incubation of the blot with the streptavidin-HRP protein and subsequent detection steps. As an alternative, use Bovine Serum Albumin (BSA) at a concentration of 3% in TBS-T 1×, or a commercial (biotin-free) blocking buffer as described in this protocol.

49. Add the appropriate antibody to the blocking buffer and incubate for 1 h at 20°C–25°C or follow the manufacturer’s instructions.
  a. Use a BirA (mutated/TurboID) antibody diluted 1:5000 in StartingBlock (TBS) Blocking Buffer to determine the accumulation of the Turbo-ID fusion protein *in planta.*
  b. Use a Streptavidin-HRP protein diluted 1:5000 in StartingBlock (TBS) Blocking Buffer to check for the presence of biotinylated proteins purified from the pull down using the streptavidin beads.
  c. Use an α-HA, α-GFP or α-V5 antibody to detect the accumulation of fusion proteins carrying these tags *in planta.* In this protocol, we used α-GFP diluted 1:1000 in StartingBlock (TBS) Blocking Buffer.
  d. Use appropriate antibody(ies) when checking for the presence of additional differently labelled interactor(s) after the pull down e.g., to verify the biotinylation of a tagged known interactor.

***Note:*** The dilutions of antibodies are based on specifications for Western blotting detection substrate used. In this protocol, we used SuperSignal West Dura.

**Figure 1:**
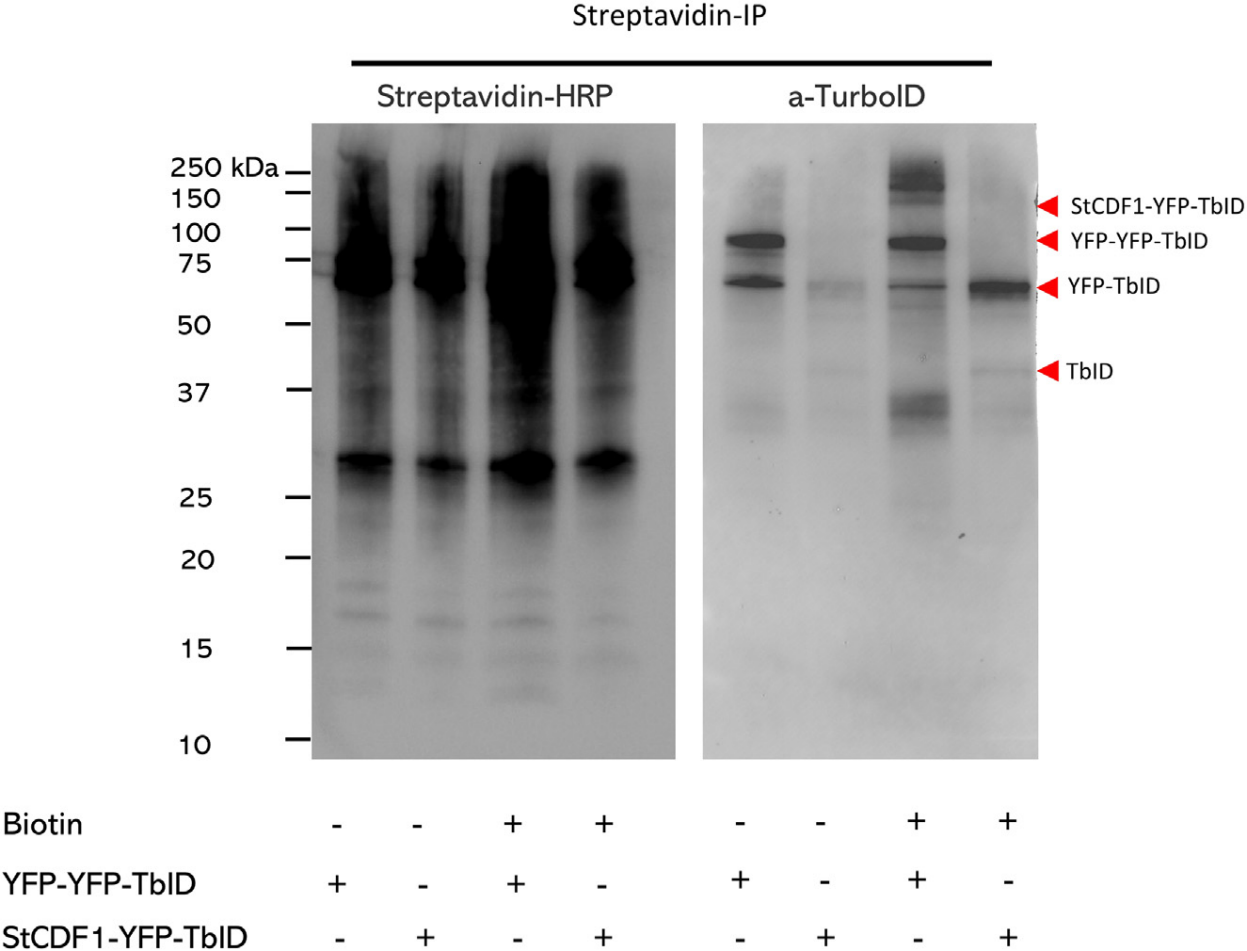
Immunoblot analysis of protein extracts obtained in step 49 Immunoblot (IB) analysis following streptavidin affinity purification of potato (CE3027) leaf material transiently transfected with StCDF1-YFP-TurboID and YFP-YFP-TurboID, which was infiltrated with 10 μM of exogenous biotin 2 h before harvest, as well as an untreated control sample. Biotinylated proteins were immunoblotted by Streptavidin-HRP (left). StCDF1-YFP-TurboID and YFP-YFP-TurboID was immunoblotted by anti-TurboID antibody (right). The red arrows represent target proteins. Expected sizes of StCDF1-YFP-TurboID and YFP-YFP-TurboID proteins are 122.4 and 99.6 kDa, respectively. In addition to full length or partial bait protein, YFP (27 kDa) and TurboID (35 kDa) free tags should be also visualized by using corresponding antibodies. In this context, partial proteins due to degradation could also be present in the Western blot. Partial proteins are: YFP-V5 (27 kDa), YFP-V5-TurboID (63 kDa), V5-TurboID (36 kDa), V5-TurboID-HA (37 kDa) and TurboID-HA (36 kDa).

50. If a secondary antibody is needed as for the BirA (mutated/TurboID) antibodies:
  a. Wash the membrane 3 times with TBS-T 1× buffer.
  b. Dilute the conjugated secondary antibody.
    ***Note:*** In this protocol, we used goat anti-rabbit IgG, HRP diluted 1:10000 in TBS-T 1× buffer.
  c. Incubate with the secondary antibody for 1 h at 20°C–25°C.
51. Wash the membrane 3 times with TBS-T 1× buffer.
52. Develop the blot with the ECL substrate of choice (Figure 1).

### Identification of the biotinylated proteins by mass spectrometry

**Timing: 1–4 weeks**

The enriched biotinylated proteins are sent to the MS facility and subsequently analyzed in a specific software for protein identification and statistical analysis.

53. Send the samples from **step 37** to the MS facility.

***Note:*** It might be required to store the sample in a specific buffer at a determined temperature before the samples are pre-processed for MS.

***Note:*** We sent our samples to MS facility in Biochemistry Department at WUR for LC-MS analysis.

***Note:*** MS facility in Biochemistry Department at WUR pre-processes the samples to perform LC-MS.^10^ The pre-processing involves cysteine reduction, tryptic digestion, and clean-up of the peptides. These steps can vary depending on the facility service.

54. Use the appropriate software for statistical analysis of the results from the LC-MS.
55. Select the proteins of interest for further studies.

## Expected outcomes

**Figure 2:**
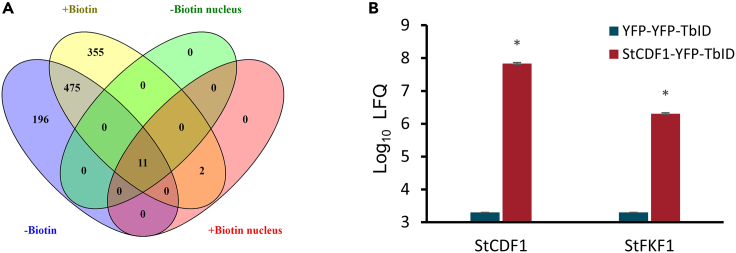
Overview of mass spectrometry results of StCDF1-YFP-TbID (A) The Venn diagram illustrates the overlaps of enriched proteins in StCDF1 samples among protein sets under different treatments, including with and without extra biotin supply, with extra biotin supply and nucleus located, and without extra biotin supply and nucleus located proteins. (B) Protein enrichment of StCDF1 and StFKF1 was analyzed in YFP-YFP-TbID and StCDF1-YFP-TbID samples (with extra biotin treatment) using mass spectrometry (MS). The data is presented as the mean of logarithm-based label-free quantification (LFQ) values ± standard error (n = 3). Statistical significance was determined using a student’s t-test (∗P < 0.05). Notably, both proteins were not detected in the YFP-YFP-TbID samples. To address this, missing values were imputed using imputation methods based on the normal distribution.

To evaluate the efficacy of the transient TurboID-based proximity-dependent labeling method in *S. tuberosum*, several criteria can be assessed. First, the fused POI should be detected in transformed leaves. In this protocol, the POI is fused with YFP and TurboID. The YPF tag allows performing a confocal study to check the subcellular localization of POI. For instance, StCDF1 is known to accumulate in the nucleus,^11^ while NbSOBIR1 is found at the plasma membrane.^12^^,^^13^ Potential candidate interactors could be also filtered out by the localization of POI after statistical filtering. Secondly, the tagged POI should be produced and accumulate in potato leaves and be able to biotinylate proximal proteins. Abundance of POI-YFP-TurboID can be checked in Western blot using YFP and TurboID antibodies in step 49. Our previous observations suggest that overexpressing tagged protein in potato always leads to the issue of the tag being cleaved off. The POI-YFP-TurboID can be degraded and lead to no full-size POI-YFP-TurboID present from immunoblot (IB), but only partials (Figure 1). The absence of the full-size band in IB does not necessarily mean no accumulation or experiment failure. Figure 2A provides an overview of the proximity labeling MS results for StCDF1-YFP-TbID. Our analysis reveals the identification of more than 10 proteins located within the nucleus based on the MS dataset, showing positive enrichment in StCDF1 samples (Figure 2B). Notably, among these proteins are StCDF1 itself, as well as a well-known interactors, StFKF1.^2^ Moreover, by comparing the MS results between applying extra biotin and without, it is noticeable that there are 2 nucleus located proteins that were enriched only when extra biotin was supplied. However, the extra biotin application also increased the total amount of labelled proteins (Figure 2A).

Western blotting can also be used to determine the efficiency of the enrichment of the biotinylated proteins, including the POI that is used as a bait fused to the TurboID enzyme, and control proteins fused to TurboID, such as YFP, GFP or GUS. The specificity of the labeling with biotin can be demonstrated using Streptavidin-HRP. The intensity of the signals can be used to estimate the labeling efficiency, which should be relatively high when the fusion protein accumulates in sufficient amounts *in planta*. It is worth noting that a low protein level of the bait can also be desirable in other plant systems such as *N. benthamiana*, as using a proper negative control will help avoid background labeling. However, we find that tagged protein yield in potato transient transformation experiments is generally lower. Furthermore, we need to consider that potato tends to cleave the tagged protein. Consequently, the enrichment of biotinylated proximal proteins from low protein level POI-YFP-TurboID is rather poor in potato transient experiments. Therefore, getting good production of POI-YFP-TurboID in potato is a key step for a successful TurboID-based PL approach.

Finally, the MS output comprises a plot of intensity that enables the identification of the biotinylated proteins in the sample. Intensities of proteins identified in the transiently expressed POI-TurboID samples are compared to the samples carrying the negative TurboID controls. Proteins that are significantly enriched in the POI-containing sample, when compared to the negative control, can be considered putative interactors of the POI. The identification of these proteins as interactors can be further validated by other methods, e.g., *in vivo* interaction assays such as yeast two-hybrid, *in vitro* assays such as co-immunoprecipitation or silencing studies (VIGS, CRISPR) followed by screening of the response (change in phenotype, loss-of-function phenotype). Moreover, the biological relevance of the putative interactors for POI functioning can be assessed by gene ontology analysis, which can reveal the biological processes or pathways in which they are involved.

Overall, the expected outcome of the transient TurboID-based proximity-dependent labeling method is a comprehensive and specific characterization of the protein interactome of the POI in *S. tuberosum*. This method has the potential to uncover novel interactors and protein complexes, thereby shedding light on the biological functions and mechanisms of action of the POI.

## Limitations

There are several limitations to this transient TurboID proximity-dependent labeling protocol for identifying PPIs in *S. tuberosum*. First, the approach of transient expression by agroinfiltration limits the experimental tissue to only leaves of potato. For detecting certain tissue-specific PPIs, stable transformed plants should be considered. Second, with the same amount of input leaf material and the same PL approach, the protein yield from potato leaf material is generally lower than that from *N. benthamiana*. For the MS analysis, this lower protein yield will not pose a major problem. However, this low protein yield could cause some difficulties concerning the western blots, for example in detecting the POI which has been fused with the TurboID enzyme. Longer exposure times for the blots, combined with using high sensitivity ECL and/or decreasing antibody dilutions should be considered to solve this problem. Third, it is important to keep in mind that this PL method allows covalent labeling with biotin of any proteins that are in the proximity of the bait POI at a particular moment. *In vivo* analysis still needs to be performed to verify an actual direct or indirect interaction between the two proteins.^9^ Moreover, the labeling radius of TurboID is estimated to be around 35 nm and the labeling ability depends on the available lysines in the proximal proteins and the availability of biotin.^14^^,^^15^^,^^16^^,^^17^

## Troubleshooting

### Problem 1

The potato genotype of interest is not amenable to *A. tumefaciens* infiltration, or the bait protein does not accumulate in the selected potato genotype.

### Potential solution

• Reduce the OD_600_ of the *A. tumefaciens* cell suspension to 0,3 or lower.
• Screen several genotypes from the same species.
• Screen additional *A. tumefaciens* strains that have a lower virulence.
• Consider stable transformation of the potato genotype of interest.

### Problem 2

The potato genotype fails to produce sufficient leaves and exhibits poor growth after four weeks of growth in the pots.

### Potential solution

• Optimize the different growth conditions for the various potato genotypes by considering their individual preferences, such as their preference for a greenhouse with natural light or artificial light.
• Change the type of pots. Some genotypes prefer a different pot size or shape compared to others.

### Problem 3

The potato genotype is difficult to perform an infiltration with the *A. tumefaciens* suspension on.

### Potential solution

• Water the plants 2 h before infiltration or spray the leaves with water mist immediately before infiltration.
• Use a needle, scalpel or P-10 pipette tips to create small wounds on the abaxial surface of the leaves before infiltration.

### Problem 4

Low overall biotinylation signal as revealed by Western blotting (step 49).

### Potential solution

There may be multiple causes of a low biotinylation signal, including.

• The endogenous biotin content of the potato leaves is not high enough for efficient labeling. Provide extra biotin (10 μM biotin, 10 mM MES, pH 8) at 1–3 h before harvesting.
• The gene encoding the TurboID fusion protein is poorly expressed. To improve the level of gene expression, modifying the promoter should be considered. In addition, codon optimizing of the open reading frame encoding the POI and TurboID could also help to improve the expression levels.
• The POI fused with the TurboID enzyme is accumulating poorly. Consider changing the site at which the TurboID is fused to the POI. The C-terminal end of StCDF1 is responsible for its stability,^2^ and fusing TurboID to the C-terminal end of StCDF1 will inhibit its degradation and thereby enhance the labeling ability.
• Consider mutating any degradation signals or motifs in the POI. For example, using the StCDF1.2 allele (increased protein stability due to missing binding site for StFKF1^2^) rather the full length wild type StCDF1 (1.1). However, keep in mind that mutating this binding motif will affect the final MS result. The interactor which mediating the degradation, such as StFKF1 will not appear in StCDF1.2 proximity labeling MS results.

### Problem 5

Unbalanced expression by the negative control-expressing plasmids and POI-expressing plasmids.

### Potential solution

• Transform plasmids to different strains of *A. tumefaciens.*

***Note:*** In this study, both negative control plasmids were transformed to the C58C1 strain of *A. tumefaciens*, and the POI-expressing plasmids were transformed to AGL1. For the same *A. tumefaciens* strain, the negative control plasmid generally resulted in a higher expression than the POI-expressing plasmid. Therefore, C58C1, which results in lower protein accumulation levels, was selected to be used for both negative controls. When compared to C58C1, the AGL1 strain can help to promote higher expression levels of the POI-containing plasmids, but also possibly induces more cell death in some potato genotypes.

### Problem 6

A protein that has previously been found to interact with the POI is not present in the dataset generated by the LC-MS experiments.

### Potential solution

• The absence of a known protein interactor does not necessarily mean that the protocol is not appropriate. For tissue-specific PPIs, stable transformation should be performed, and only the target tissue be harvested. If the expression of potential interactors follows the circadian rhythm, adjust the sample harvesting time.

### Problem 7

Background noise due to endogenous biotinylated proteins, unrelated to the presence of the POI fused to TurboID.

### Potential solution

• Including a second control: using POI fused only with YFP and without TurboID enzyme.

### Problem 8

The LC-MS analysis of the sample for which the POI fused to TurboID was expressed, produced limited results, with only a small number of biotinylated proteins showing significant enrichment compared to the negative control sample.

### Potential solution

• Perform targeted protein isolation based on POI localization.
• Add proteasome inhibitor (MG-132).

## Resource availability

### Lead contact

Further information and requests for resources and reagents should be directed to and will be fulfilled by the lead contact, Vivianne Vleeshouwers (vivianne.vleeshouwers@wur.nl).

### Materials availability

Potato genotypes and described plasmids are available upon request from the lead contact.

### Data and code availability

All relevant data are available from the lead contact upon request. There are no restrictions on data availability.
